# The CAIDE Risk Score Predicts Mild Cognitive Impairment in an Urban Indian Cohort Study of Aging

**DOI:** 10.1002/alz70857_107654

**Published:** 2025-12-25

**Authors:** Abhishek Mensegere Lingegodwa, Albert Stezin, Jonas S Sundarakumar, Thomas Gregor Issac

**Affiliations:** ^1^ Centre for Brain Research, Indian Institute of Science, Bangalore, Karnataka, India

## Abstract

**Background:**

The Cardiovascular Risk Factors, Aging, and Incidence of Dementia (CAIDE) risk score is a validated tool to estimate the risk of dementia using midlife risk factors and is the most well‐known for dementia prediction. This study aimed to investigate the association between high‐risk CAIDE group membership and the subsequent development of mild cognitive impairment (MCI) among community‐dwelling older Indian adults.

**Method:**

Data were analyzed from the TATA Longitudinal Study of Aging. Participants were categorized into high‐risk (>9) and low‐risk (9) groups based on their CAIDE scores. MCI was diagnosed using a clinical dementia rating scale (CDR = 0.5). Binary logistic regression was employed to assess the odds of developing MCI (dependent variable) after one year of follow‐up in the high‐risk CAIDE group compared to the low‐risk group. Additionally, we used the CAIDE score as a continuous variable in the binary logistic regression to estimate the risk as the score increased. Lastly, we ran multiple linear regression to estimate the impact of cardiovascular risk on cognition among participants who did not develop MCI.

**Result:**

In a sample of 939 non‐dementia participants at baseline 48 (3.5%) developed MCI after 1 year. Individuals in the high‐risk CAIDE group had significantly increased odds of developing MCI compared to their low‐risk counterparts (Odds Ratio = 2.58, 95% CI [1.34, 4.94], *p* =  0.004). Every one‐point increase in CAIDE score increased the odds of developing MCI by 33% (Exp β = 1.33, 95% CI [1.13, 1.56], *p* < 0.001). Furthermore, in the participants who did not develop MCI in the follow‐up visit, the high‐risk CAIDE group performed poorly in ACE total scores in the follow‐up visit (β = ‐1.728, 95% CI [‐2.544, ‐0.912], *p* < 0.001).

**Conclusion:**

This study provides evidence that individuals identified as high‐risk according to the CAIDE criteria may be at an elevated risk of developing MCI. These findings underscore the potential shared pathophysiological pathways between cardiovascular and cognitive health and emphasize the importance of early identification and intervention for individuals at high risk for both CVD and cognitive decline.

